# Effect of Distraction Intervention for Needle-Related Pain and Distress in Children: A Systematic Review and Meta-Analysis

**DOI:** 10.3390/ijerph18179159

**Published:** 2021-08-31

**Authors:** Mi-Kyoung Cho, Mi-Young Choi

**Affiliations:** Department of Nursing Science, Chungbuk National University, Cheongju 28644, Korea; ciamkcho@gmail.com

**Keywords:** child, distraction intervention, distress, meta-analysis, needle-related pain, systematic review

## Abstract

A systematic review and meta-analysis conducted to evaluate the combined effect of distraction intervention for needle-related pain in order to provide the basis for developing an effective nursing intervention for children. We searched three electronic databases, PubMed, Embase, and CINAHL, for original articles published in the period from 1 January 2011 to 31 July 2019. In addition, a manual search was performed on the basis of references in the literature and the references of the articles in pursuit of comprehensive data until 10 December 2019. Meta-analysis was performed by the synthesis of the effect size, homogeneity, heterogeneity, and trim-and-fill method using MIX 2.0 Pro. Well-planned RCTs, single-center studies, high-quality studies, participants older than 10 years of age, and visual and clown distraction interventions were effective for needle-related pain and distress management among children. The results showed evidence supporting the effect of distraction interventions for children’s needle-related pain and distress. Through this review, strategies were identified to design better interventions to improve the outcomes.

## 1. Introduction

Several international pain-related expert groups, including the World Health Organization (WHO), contend that optimally effective pain management is a fundamental human right and good and ethical practice [1,2]. Therefore, healthcare providers, including pediatric nurses, have an ethical obligation and responsibility to use the up-to-date scientific ground to relieve any pain and fear that children may experience during treatment [3].

Both healthy and medically ill children may have difficulty with needle-related procedures [4]. Vaccination, blood collection, and intravascular injection are treatment procedures causing pain and fear that children may most frequently experience [4,5,6]. They may have such painful experiences in diverse places, including public health clinics and hospitals [1]. As children grow and receive routine vaccinations, they are regularly exposed to injections and pain. The current recommendations are that healthy children should get 20 to 30 vaccinations before the age of 18 years [7] and the majority of them are given within the first six years after birth [8]. Children with acute or chronic diseases frequently undergo needle-related procedures to determine their condition [9]. Children can have greater difficulty in understanding, accurately expressing, or overcoming the distress and pain they experience during the treatment process. Even a minimal medical procedure can arouse meaningful pain and distress among some children [10]. Needle-related procedures induce anxiety and fear in children due to the related pain and are also associated with negative emotions and feelings [11,12]. In addition, they often involve significant pain and distress for both children and parents [9]. The response to pain is a learned behavior and needle phobia can have many different causes; previous research reported that it was associated with any negative experience of pain [10]. Children remember the past painful experiences, and the negative memory of pain or fear caused by poor management during treatment can affect the subsequent treatment, consequently having an adverse long-term impact on future responses to pain [13]. It is; therefore, necessary to make an intervention for children that would properly, immediately, and effectively control needle-related pain and distress in nursing practice.

Because it is very important and difficult to evaluate and manage children’s pain, many hospitals regard pain as the fifth vital sign [14,15,16,17]. It has been suggested that pain be managed to give children the optimal comfort before, during, and after pain-causing treatments [2]. Although pain management for children has continually received attention for 20 years and its importance has been recognized, and the understanding of children’s pain has grown and considerable knowledge about its management has been accumulated through scientific exploration, many children still receive improper pain management, along with poor specific practice [1,2]. 

With the report that children’s pain and fear associated with needle insertion can adversely affect the process of treatment, various interventions have recently been introduced to relieve them. Albeit it is necessary to apply interventions that can effectively reduce pain and distress in children, it is difficult to apply them in clinical practice when special preparation and training and complicated procedures are required. As distraction techniques are often applied by nurses and parents and can relieve pain in children during treatment, they have recently attracted attention as practical nursing interventions [7]. 

Distraction intervention is defined as a cognitive or behavioral strategy to relieve pain, stress, and anxiety by distracting children from a harmful stimulus to what they do or something attractive through a non-pharmacological method of pain management [18]. Literature review presents evidence that distraction intervention is a simple and effective non-pharmacological pain management strategy for both healthcare providers and parents to relieve procedural pain in children with ease and at a low cost [5,17].

Pain management needs to be provided on a scientific ground, not based on personal opinions or beliefs; every healthcare provider needs to provide optimal pain management [2]. Nurses who apply any non-pharmacological intervention to relieve procedural pain, including distraction intervention, need to determine if it is appropriate and effective [3]. Some researchers presented a direction for further research and clinical practice related to distraction techniques through a critical review of the research on distraction interventions applied in children to relieve procedural pain [5]. Birnie et al. [7] performed a systematic review and meta-analysis related to the efficacy of distraction and hypnosis for relieving needle-related pain and distress in children and adolescents. However, there has been no meta-analysis of the effectiveness of distraction interventions for relieving needle-related pain and distress in children. Therefore, in this study, we performed a comprehensive comparative analysis of the effectiveness of distraction interventions to relieve needle-related pain and distress in children of diverse backgrounds to identify the most effective intervention program and suggest a direction for effective distraction interventions required to control.

## 2. The Review

### 2.1. Aims

This study aimed to estimate the overall effect size of distraction interventions on needle-related pain and distress in children and provide a ground for developing a nursing intervention that would effectively relieve needle-related pain and distress in children.

### 2.2. Design

We performed a systematic and comprehensive review and a meta-analysis to determine the effects of distraction interventions on needle-related pain and distress in children. The study was performed according to the systematic consideration handbook presented by Cochrane Collaboration [19] and the systematic consideration reporting guidelines presented in Systematic Reviews and Meta-Analysis by Littell, Corcoran, and Pillai [20].

### 2.3. Search Methods

Articles were first searched based on the criteria of population, intervention, comparison, outcome, and study design (PICO-SD). The population was limited to children, intervention to distraction intervention for relieving needle-related pain and distress, comparison to patients given usual care, outcome to needle-related pain and distress, and study design to randomized controlled trials (RCTs) and studies with a quasi-experimental design. An electronic database search was performed for national and international studies according to the inclusion and exclusion criteria to choose articles published in English that showed specific statistics of the intervention effects, including the mean and standard deviation and the sample number.

Three electronic databases—PubMed, Embase, and CINAHL (Supplementary Table S1)—were used to search for articles published from 1 January 2011 to 31 July 2019. In addition, a manual search was performed based on references in the literature and the references of the articles in pursuit of comprehensive data until 10 December 2019. Medical Subject Heading (MesH) terms, synonyms, and related terms for expressing “child” or “children”, “distraction” or “distraction intervention”, “pain” or “needle-related pain” or “venipuncture pain” or “immunization pain” according to PICO were identified in PubMed and adapted to the properties of each database by using such search functions as MesH terms, text words, logical operators, and truncation search properly.

### 2.4. Search Outcomes

**Figure 1:**
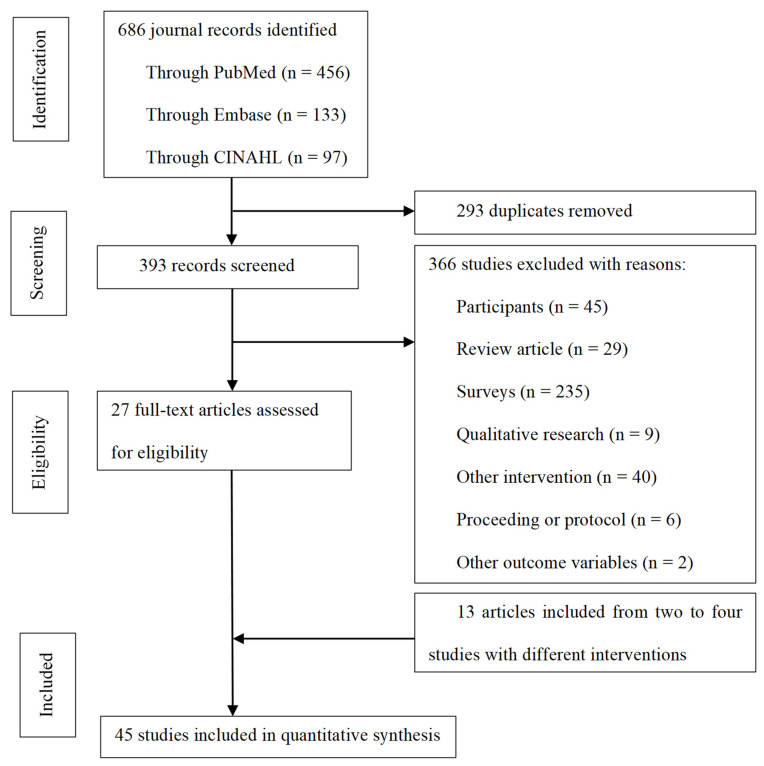
Search outcomes.

Of the total 686 screened articles (456 from PubMed, 133 from the Embase, and 97 from CINAHL), 27 articles were finally included based on the exclusion criteria for overlapping, population, study design, intervention, and variables. Of these, 13 articles with two experimental groups were included in the analysis, with the interventions in each group separated from each other. As a result, a total of 45 studies were included in this analysis (Figure 1).

### 2.5. Quality Appraisal

The Checklist for Randomized Controlled Trials and the Checklist for Quasi-Experimental Studies in the Joanna Briggs Institute of Critical Appraisal Tools [21] were used to evaluate the quality of the selected RCTs and quasi-experimental studies, respectively, and to access the risk of bias of individual studies. As a pilot test, two researchers independently evaluated a total of four articles—two RCTs and two quasi-experimental studies—to investigate whether the quality evaluation tools had the same score for each item. For the items with inconsistent scores, the agreement was reached through discussion to clarify the evaluation criteria, followed by independent quality evaluation and comparison of the scores for the quality evaluation items in the final articles. The quality evaluation items for RCTs were as follows: random assignment, allocation concealment, treatment groups similarity, blinding of participants, blinding of treatment delivering, blinding of outcome assessor, similar treatment, follow-up complete and if not, adequately described and analyzed, the intention-to-treat analysis, same way of outcome measures, reliable way of outcome measures, appropriate statistical analysis, and appropriateness of the trial design. The score for each quality evaluation item ranged from 0 (No, Unclear) to 1 (Yes), with a total score of 13. The quality evaluation items for quasi-experimental studies were as follows: clarity of the cause and outcome effects, treatment groups similarity, similar treatment, comparison of the treated group, multiple measures, follow-up complete and if not, adequately described and analyzed, same way of outcome measures, reliable way of outcome measures, and appropriate statistical analysis. The score for each quality evaluation item ranged from 0 (No, Unclear) to 1 (Yes), with a total score of 9. The absence of tables or contents in the text, even if described in the abstract, was scored 0.

### 2.6. Data Abstraction

Throughout the course of data screening and collection, two researchers independently reviewed each study included in the analysis. The EXCEL program was used to exclude overlapping articles. Next, titles and abstracts were reviewed to identify articles that met the inclusion criteria; if it was difficult to determine if they met the criteria on the basis of titles or abstracts alone, the original text was referenced to determine inclusion. The bibliographical information of every article was coded in the EXCEL program and managed in the identical way, with the excluded articles recorded by stages. For the final articles, authors, publication year, country, number of centers, population size, participants’ age, Institutional Review Board (IRB) approval status, funding status, research design, intervention type and characteristics, intervention in the control group, outcome variables, and quality evaluation scores were collected and recorded in the coding table.

### 2.7. Synthesis

The characteristics of the studies were presented as frequencies or the mean and standard deviation. MIX 2.0 Pro (Ver. 2.0.1.6, BiostatXL, 2017, BiostatXL, CA, USA) was used for statistical analysis of the method of combining the effect size. For the effect size, standardized mean difference and 95% confidence intervals (CIs) were used for the same outcome variable and the weight of each effect size was estimated using the inverse number of variances [22]. The random effects model for resetting weighted values, considering the variability among the participants in different studies and inter-study heterogeneity, was used to estimate the overall effect. A sub-analysis was performed on the basis of the participants’ age and the following study characteristics: number of centers, IRB approval and funding status, study design, intervention type, and scores in the quality evaluation. For the heterogeneity, Higgin’s I^2^ for the actual variance of the observed total variance or the interobserver variance rate was estimated [23], with I^2^ > 50% regarded as indicator of heterogeneity [19]. The funnel plot was used to modify the standardized mean difference to test the studies for publication bias [24].

## 3. Results

### 3.1. Study Characteristics

There were 14 studies published before 2015 and 31 after 2015; six in East Asian countries, such as India and Taiwan, 12 in American countries, such as the United States, Canada, and Brazil, and 27 in other countries. There were 40 single-center studies and 36 had a population of 50 to less than 300 persons. In 41studies, the mean age of the population was <10 years and four studies (study ID: 28, 29, 30, 33) had participants aged ≥10 years. Forty-one studies had IRB approval and 15 were funded. There were 41 RCTs, including two crossover RCTs, and four quasi-experimental studies. Regarding distraction intervention types, visual distraction was used in 19 studies, audial distraction in seven, clowns in two, cognitive behavioral therapy in two, multiple methods, including touch and acupressure, in 13, and local anesthesia in two studies. Thirty-eight studies involved no intervention or usual care given by parents in the control group. Characteristics of the included studies are presented in Table 1.

### 3.2. Methodological Quality

For the quality evaluation, two RCTs and two quasi-experimental studies among the final 45 studies were included in the pilot test for scores. There was 90.5% consistency, and it was agreed that inconsistent items would be scored 0 if the homogeneity was “not tabulated in the results of the text though described in the abstract”. In the methodological quality evaluation, 41 RCTs scored 9.05 (range = 7–12) and four quasi-experimental studies scored 7.00 (range = 5–9). As for the qualitative level of the studies, the conclusion had no possibility of being changed.

### 3.3. Effect of Distraction Intervention on Needle-Related Pain in Children

**Figure 2:**
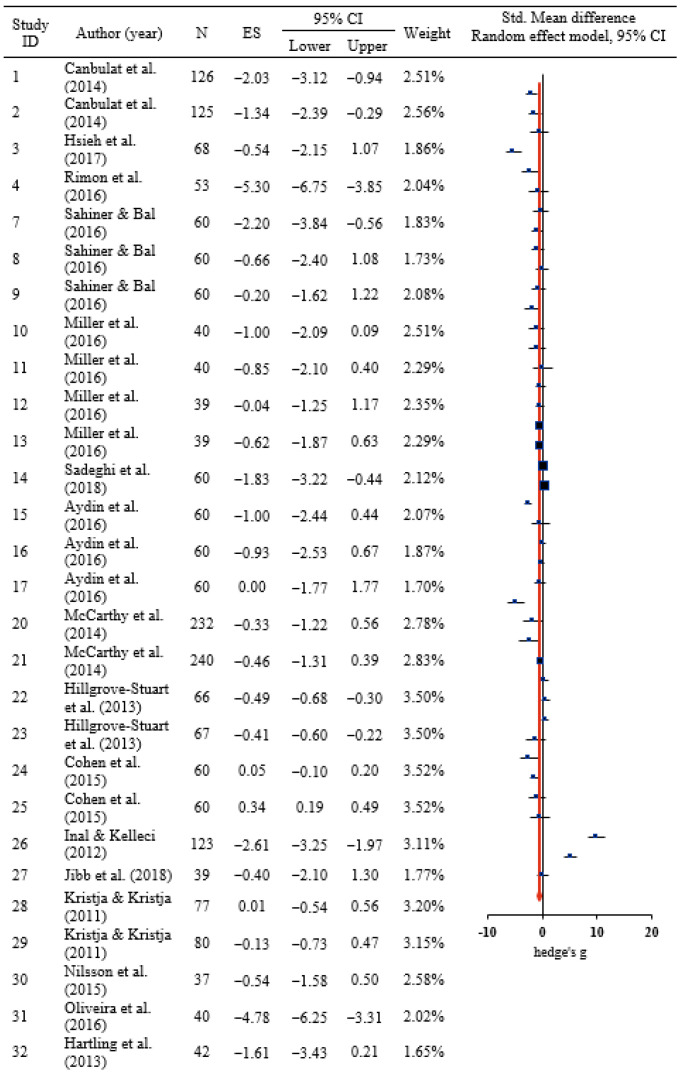

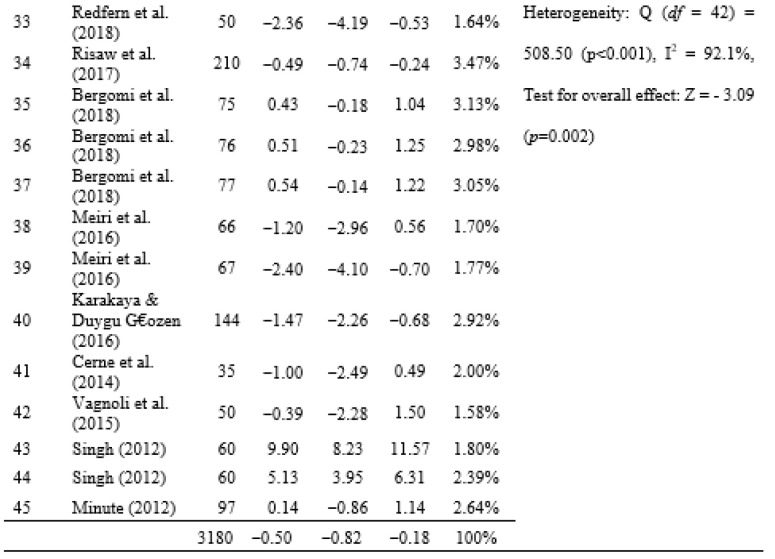
The effect of distract intervention on needle-related pain of the child. ES: Effect size, CI = Confidence interval.

In the 41 studies, the mean of the differences between the pre-test and the post-test in both groups, the standard deviation of the differences, and the sample size were used to estimate standardized mean differences, which were presented in a synthesis forest plot (Figure 2). Since the characteristics of the included study were heterogeneous, two methods were used to calculate the combined effect size in this study. First, for the calculation of the combined effect size in this study, hedge’s g was weighted the standard deviation by its sample size for corrected effect size, because Cohen’s *d* tends to overestimate the population variance, especially for small samples (n < 20) [25]. Second, the random effect model was used rather than the fixed effects model to analyze the random error of the effect sizes of each study and the error according to the characteristics of the study [26].

For the children, the overall effects of needle-related pain were at the medium level of −0.50 (95% CI = −0.82 to −0.18) and the needle-related pain decreased significantly after distraction intervention (Z = −3.09, *p* = 0.002). The effect size was highly heterogeneous: I^2^ = 92.1% [24]. Given that exploratory explanation of the background for the heterogeneity of the effect size was required, a sub-analysis was performed on the basis of the participants’ age and the following study characteristics: number of centers, IRB approval and funding status, study design, intervention type, and scores in the quality evaluation (Table 2). 

For the 26 studies with participants aged ≥10 years, the overall effects were at a high level of –1.07 (95% CI = −1.60 to −0.53) and the needle-related pain decreased significantly after distraction intervention (Z = −3.92, *p* < 0.001). For the 38 single-center studies, the overall effects were at a medium level of −0.51 (95% CI = −0.85 to −0.17) and were statistically significant (Z = −2.96, *p* = 0.003). For the 37 studies without IRB approval, the overall effects were at a medium level of –0.64 (95% CI = −0.89 to −0.39) and the needle-related pain decreased significantly after distraction intervention (Z = −5.04, *p* < 0.001). For the 15 studies with funding, the overall effects were at a high level of –0.83 (95% CI = −1.20 to −0.46) and the needle-related pain decreased significantly after distraction intervention (Z = −4.41, *p* < 0.001).

For the total 37 RCTs, including the two crossover RCTs, the overall effects were at a medium level of −0.61 (95% CI = −0.92 to −0.30) and the needle-related pain decreased significantly after distraction intervention (Z = −3.85, *p* < 0.001). While the overall effects of multiple distraction methods, including audial, cognitive, and touch and acupressure, were statistically insignificant, those of the visual distraction method was at a high level of −0.76 (95% CI = −1.15 to −0.37), which was statistically significant (Z = −3.86, *p* < 0.001). The overall effects of clowns were at a high level of −3.64 (95% CI = −4.76 to −2.52), which was statistically significant (Z = −6.36, *p* < 0.001). 

For the 17 studies with a score ≥10 in the quality evaluation, the overall effects were at a medium level of −0.65 (95% CI = −0.98 to −0.32) and the needle-related pain decreased significantly after distraction intervention (Z = −3.84, *p* < 0.001).

### 3.4. Effect of Distraction Intervention on Needle-Related Distress in Children

In the 45 studies, besides needle-related pain in children as a principal variable, observer pain was measured in parents and nurses, anxiety in children, parents, and nurses, and blood cortisol in children (Table 3). Twenty-three studies involved observer pain (parents), 28 observer pain (nurses), three child-reported anxiety, and five cortisol measurement; the overall effects of these variables were statistically insignificant after distraction intervention. For the 15 studies related to observer anxiety (parents), the overall effects were at a medium level of −0.68 (95% CI = −1.13 to −0.23) and observer anxiety (parents) decreased significantly after distraction intervention (Z = −2.96, *p* = 0.003). For the 14 studies related to observer anxiety (nurses), the overall effects were at a high level of −1.03 (95% CI = −1.67 to −0.40) and observer anxiety (nurses) decreased significantly after distraction intervention (Z = −3.20, *p* = 0.001).

### 3.5. Publication Bias Analysis

**Figure 3:**
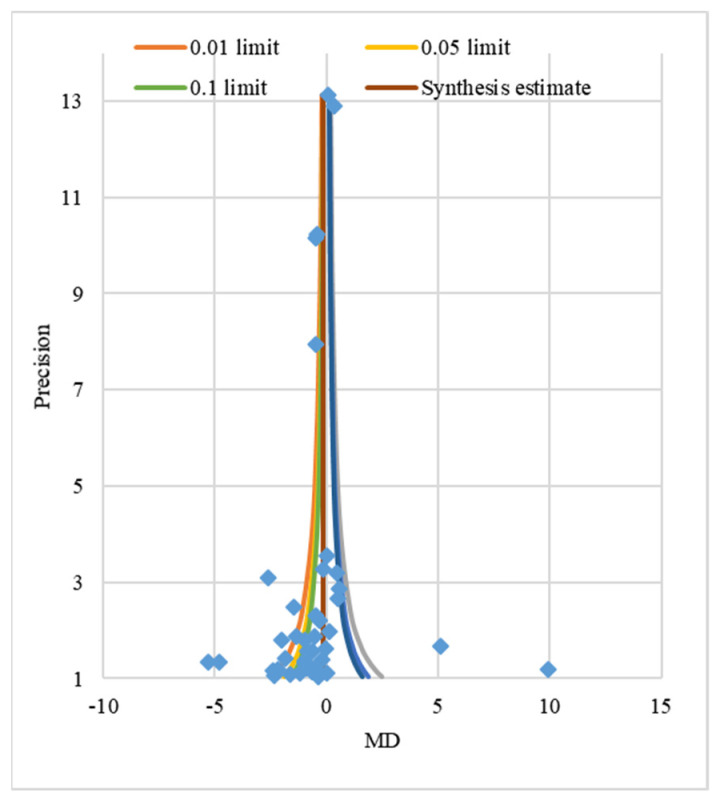
Publication bias analysis.

In the publication bias analysis for validation, funnel plot analysis was first performed, as usually recommended, to identify publication biases [20]. As seen in Figure 3, the effect size was visually symmetrical in a funnel shape. Nfs (fail-safe number), one of the criteria for reliability of meta-analysis results, was estimated to secure the reliability of the results. Nfs is a bias that can occur when the mean effect size is only based on the results of the published article without unpublished results, and Orwin’s (1983) method was applied in this study. For the 41 studies concerning needle-related pain in children after distraction intervention, the combined effect size hedge’s g = −0.50 and Cohen’s (1977) small effect size d = 0.2 were used to estimate the Nfs, and 61.5 additional studies were required to reduce the effect size to d = 0.2. Based on this finding, it cannot be said that the analyzed studies had no publication error, but the errors were not so significant as to reverse the entire results (Figure 3).

## 4. Discussion

A total of 45 studies published from 2011 to 2019 were included in the meta-analysis for the effect size of distraction intervention on needle-related pain and distress in children. The results can be discussed as follows. 

Of the 45 studies, 32 (69.6%) were published after 2015 and the majority of them were published in the United States, Canada, and Brazil (12; 26.1%). Twenty-six articles on distraction and 13 on hypnosis for needle-related pain and distress in children and adolescents [7] indicated relatively strong effects. We analyzed the effects of distraction intervention alone, excluding those of hypnosis, among psychological interventions. From the results, the research on distraction intervention had increased after 2015. Distraction intervention still failed to be used in many countries. This supports the finding that despite the far-reaching extension of knowledge about pain management and the development of effective ground-based strategies, pain management using distraction intervention is not positively applied in clinical practice and there are clear differences between the knowledge about pain management and practice [2,27].

Of the 45 studies, 41 (89.1%) were conducted in participants aged <10 years on average and 17 (37.0%) had participants aged ≥10 years. This implies that for the study design, consideration was given to the fact that young children might be vulnerable to fear of needle insertion. However, it is necessary to pay attention to the decrease of the overall effects in children aged ≥10 years in the subgroup analysis for needle-related pain. Since children aged ≥10 years may experience needle-related pain and distress in various settings, it is necessary to conduct an RCT on distraction intervention in this population.

The meta-analysis found that the overall effects of needle-related pain were at a medium level and that the needle-related pain and distress decreased significantly after distraction intervention. Regarding the type of distraction interventions, visual distraction and clown programs were effective in relieving needle-related pain and distress; therefore, it is necessary to actively develop methods of relieving needle-related pain and distress taking these intervention types into account. There are diverse settings and methods of using distraction intervention [9]. Recently, some researchers successfully used a humanoid robot as a cognitive-behavioral intervention to relieve needle-related pain and distress during annual flu vaccination [28]. This implies the development of applicable technology for new distraction interventions to relieve needle-related pain and distress. It is necessary to accumulate the effects of such interventions as evidence through strict experimental research and identify a way of applying it actively in practice. 

The RCTs that were conducted at a single center and scored ≥10 in the quality evaluation showed medium to high levels of effects, which were statistically significant. Journal editors have recently tried to apply diverse methods for securing the quality and transparency of studies. One of the methods is to ensure that researchers comply with the guidelines for reporting results; the items in the guidelines are consistent with those in the quality evaluation [29]. Evidence-based nursing requires that the most valid, reliable, high-quality results obtained using scientific methods be applied in practice; to achieve this, researchers should report their results according to the relevant reporting guidelines [30]. Therefore, considering each item in the reporting guidelines according to the study design from the stage of study planning to the writing of an article is expected to further improve the quality of studies. 

In the 45 studies besides needle-related pain directly measured in children, observer pain and anxiety were measured in parents and nurses. After distraction intervention, the overall effects on needle-related pain and observer pain were statistically insignificant. Similarly, Uman et al. [17] failed to get clear effects on distress. In contrast, observer anxiety decreased significantly at medium and high levels in parents and nurses. When a child experiences distress, the parent and the healthcare provider may often feel anxious, helpless, and guilty, accept the procedure with greater difficulty, and experience stress. In many cases, children experiencing needle-related pain and distress are given distraction intervention by their parents and the nurse as a healthcare provider and their emotions can be transmitted to the children [9]. Recently, Birnie et al. [9] found that distraction positively affected self-reported pain and distress, observer-reported pain and distress, the behavioral measures of distress, and the heart rate. In this respect, the finding of this study that distraction intervention was significantly effective in relieving anxiety in parents and nurses as observers is very meaningful. It is necessary to find a way of relieving distress in parents and nurses while developing distraction interventions to relieve needle-related pain and distress in children through repetitive research. Since children’s age, developmental level, temperament, and treatment type can affect their ability to cope with the procedural course, it is necessary to pay more attention to individual children’s preferences and disposition in pursuit of optimal results. Further research should be conducted to examine distress following distraction interventions appropriate for children’s characteristics [5,27]. Another research can be conducted to compare needle-related pain and distress by stages because it can differ from distraction interventions before, during, and after a needle-related procedure.

This study has the limitations of a meta-analysis; it is difficult to include unpublished articles and those not retrieved in the analysis. Because some of the included studies were narrow in scope, there were limitations in generalizing the results. It was a necessary process for planning new interventions in the future by analyzing various interventions, but the low statistical power of the meta-analysis results obtained for some variables of the interventions analyzed was. Furthermore, articles not written in English were excluded, which limits the interpretation. Other limitations are that few studies were conducted in older children or adolescents and that limited information about the distraction intervention procedure was presented for other researchers to conduct repeated research and was insufficient to solve any problems with the usability of distraction intervention in clinical practice. In addition, no studies have been conducted to determine whether applying distraction interventions was effective in terms of time and costs.

## 5. Conclusions

This study aimed to determine the effect size of distraction intervention on needle-related pain and distress in children, analyze the contents of the intervention, and present a direction for its practical application and further research through a meta-analysis of 45 studies published from 2011 to 2019. Despite several limitations of the previous studies, distraction intervention was significantly effective in relieving needle-related pain and distress in children. Well-planned RCTs conducted at a single-center, high-quality study, participants older than 10 years of age, and visual and clown distraction interventions were effective for needle-related pain and distress management among children. Based on these results, the following suggestions can be made:

First, it is necessary to examine the effects of distraction intervention on needle-related pain and distress more scientifically and increase intervention research to expand it into the area of nursing.

Second, it is necessary to apply it to various pediatric nursing settings with various types of longer intervention based on the theoretical framework; randomized experimental study, which is a study design effective in applying the results, is required. Methodological strictness is required in terms of study design, implementation, and reporting.

Third, it is necessary to provide education and training to parents and healthcare professionals, including nurses, to narrow the gap between theories and practice concerning distraction intervention.

## Figures and Tables

**Table 1:** Characteristics of the included studies.

Study ID	Author (Year)	Country	Center	Procedure (Sample Size)	Age (Year)	IRB	Fund	Design	Type of Intervention (Characteristics)	Control	Outcome	Quality Assessment
1	Canbulat et al. (2014)	Turkey	1 hospital	Phlebotomy (n = 126), E: 63, C: 63	7–11 (8.8 ± 1.5)	Yes	Yes	RCT	Visual distraction (distraction cards)	No intervention	Pain: WB-FACES, Anxiety: CFS	9
2	Canbulat et al. (2014)	Turkey	1 hospital	Phlebotomy (n= 125), E: 62, C: 63	7–11 (8.8 ± 1.5)	Yes	Yes	RCT	Visual distraction (Kaleidoscope)	No intervention	Pain: WB-FACES (0–10), Anxiety: CFS (0–4)	9
3	Hsieh et al. (2017)	Taiwan	1 hospital	IV (n = 68), E: 35, C: 33	6–12 (E:8.3 ± 1.6, C:7.8 ± 1.5)	Yes	No	Quasi	Cognitive-behavioral program (pre: Educational photo book 10–15 min, during: Favorite music video 5–10 min (40–60 dB))	Routine care	Pain: NRS (0–10)	9
4	Rimon et al. (2016)	Israel	1 hospital	IV (n = 53), E: 29, C: 24	2–15 (E:5.6 ± 2.8, C:6.9 ± 3.4)	No	Yes	RCT	Medical clown (pre: Distraction via humor, during: Distraction via humor 15 min)	Parent regular distraction and comfort	Pain: VAS, FPS-R, S-cortisol	7
5	Kuo et al. (2016)	Taiwan	1 medical center	IV (n = 184), E: 92, C: 92	3–7 (E:4.5, C: 4.51)	Yes	No	RCT	Visual distraction (story book reading)	Oral instruction	OSBD-R (1–4)	8
6	Kuo et al. (2016)	Taiwan	1 medical center	IV (n = 184), E: 92, C: 92	3–7 (E:4.5, C:4.51)	Yes	No	RCT	Visual distraction (cartoon viewing (iPad))	Oral instruction	OSBD-R (1–4)	8
7	Sahiner and Bal (2016)	Turkey	1 hospital	Phlebotomy (n = 60), E: 30, C: 30	6–12 (9.1 ± 1.6)	Yes	No	RCT	Visual distraction (distraction cards)	Routine care	Pain: WB-FACES, Anxiety: CFS	11
8	Sahiner and Bal (2016)	Turkey	1 hospital	60 children for Phlebotomy (n = 60), E: 30, C: 30	6–12 (9.1 ± 1.6)	Yes	No	RCT	Audial distraction (music playback)	Routine care	Pain: WB-FACES, Anxiety: CFS	11
9	Sahiner and Bal (2016)	Turkey	1 hospital	Phlebotomy (n = 60), E: 30, C: 30	6–12 (9.1 ± 1.6)	Yes	No	RCT	Touch distraction (balloon inflation)	Routine care	Pain: WB-FACES, Anxiety: CFS	11
10	Miller et al. (2016)	Australia	1 hospital	IV (n = 40), E: 20, C: 20	3–12 (E:8.1 ± 3.0, C:7.0 ± 2.3)	Yes	No	RCT	Visual distraction (PSP: Sony handheld PSP, age- appropriate games)	Standard distraction	Pain: WB-FACES, VAS (0–10), FLACC (0–10)	11
11	Miller et al. (2016)	Australia	1 hospital	IV (n = 40), E: 20, C: 20	3–12 (E:8.1 ± 3.0, C:6.4 ± 2.9)	Yes	No	RCT	Multisensory stimuli (animated visual content, sound) and vibration; Ditto-D: PSP + interactive games)	Standard distraction	Pain: WB-FACES, VAS (0–10), FLACC (0–10)	11
12	Miller et al. (2016)	Australia	1 hospital	IV (n = 39), E: 19, C: 20	3–12 (E: 8.1 ± 3.0, C: 6.2 ± 2.8)	Yes	No	RCT	Multisensory stimuli (animated visual content, sound) and vibration; Ditto-PP: interactive procedural preparation story)	Standard distraction	Pain: WB-FACES, VAS (0–10), FLACC (0–10)	11
13	Miller et al. (2016)	Australia	1 hospital	IV (n = 39), E: 19, C: 20	3–12 (E:8.1 ± 3.0, C:6.0 ± 2.3)	Yes	No	RCT	Multisensory stimuli (animated visual content, sound) and vibration; Ditto-C: story + interactive stories and games)	Standard distraction	Pain: WB-FACES, VAS (0–10), FLACC (0–10)	11
14	Sadeghi et al. (2018)	Iran	1 hospital	IV (n = 60), E: 30, C: 30	4-6	No	Yes	Quasi	Touch distraction (press a soft ball with the opposite hand	No intervention	Pain: WB-FACES	7
15	Aydin et al. (2016)	Turkey	1 hospital	Phlebotomy (n = 60), E: 30, C: 30	7–12 (E:9.3 ± 1.8, C:9.9 ± 2.0)	Yes	No	RCT	Visual distraction (distraction cards)	Routine care	Pain: WB-FACES, Anxiety: CFS	9
16	Aydin et al. (2016)	Turkey	1 hospital	Phlebotomy (n = 60), E: 30, C: 30	7–12 (E:9.7 ± 2.2, C:9.9 ± 2.0)	Yes	No	RCT	Touch distraction (soft ball)	Routine care	Pain: WB-FACES, Anxiety: CFS	9
17	Aydin et al. (2016)	Turkey	1 hospital	Phlebotomy (n = 60), E: 30, C: 30	7–12 (E:9.7 ± 2.4, C:9.9 ± 2.0)	Yes	No	RCT	Touch distraction (balloon inflation)	Routine care	Pain: WB-FACES, Anxiety: CFS	9
18	Pour et al. (2017)	Iran	2 hospital	Phlebotomy (n = 80), E: 40, C: 40	6–12 (8.0 ± 1.8)	Yes	No	RCT	Local anesthesia (2g EMLA cream, 45 min)	Routine care	Pain: FLACC (0–10)	8
19	Pour et al. (2017)	Iran	2 hospital	Phlebotomy (n = 80), E: 40, C: 40	6–12 (8.0 ± 1.8)	Yes	No	RCT	Acupressure (Yintang (extra 1) and Laogong (P-8))	Routine care	Pain: FLACC (0–11)	8
20	McCarthy et al. (2014)	USA	3 hospitals	IV (n = 232), E: 116, C: 116	4–10 (7.3 ± 1.9)	Yes	Yes	RCT	Visual distraction (enhanced: RA distraction coaching)	Patent directed distraction	Pain: Oucher (0–10), PRCD (1–7), OSBD-R (1–4), Salivary cortisol	8
21	McCarthy et al. (2014)	USA	3 hospitals	IV (n = 240), E: 124, C: 116	4–10 (7.3 ± 1.9)	Yes	Yes	RCT	Visual distraction (professional: RA distraction support)	Patent directed distraction	Pain: 23 Oucher 24 (0–10), PRCD25 (1–7), OSBD-26R (1–4), Salivary cortisol	8
22	Hillgrove-Stuart et al. (2013)	Canada	1 clinic	IV (n = 66), E: 32, C: 34	1–2 (1.3 ± 0.2)	Yes	Yes	RCT	Touch distraction (parent directed distraction)	Routine care	Pain: M29BPS (0–10), 30MAISD	11
23	Hillgrove-Stuart et al. (2013)	Canada	1 clinic	IV (n = 67), E: 33, C: 34	1–2 (1.3 ± 0.2)	Yes	Yes	RCT	Touch distraction (RA directed distraction)	Routine care	Pain: MBPS (0–10), MAISD	11
24	Cohen et al. (2015)	USA	1 hospital	Immunization (n = 60), E: 30, C: 30	4–6.5 (4.8 ± 0.8)	Yes	Yes	RCT	Visual distraction (age- appropriate movie+ parent teaching program)	Routine care	Pain: CAPS, parent knowledge, parent behavior, children behavior	11
25	Cohen et al. (2015)	USA	1 hospital	Immunization (n = 60), E: 30, C: 30	4–6.5 (4.8 ± 0.8)	Yes	Yes	RCT	Visual distraction+ education (age-appropriate movie)	Routine care	Pain: CAPS, parent knowledge, parent behavior, children behavior	11
26	Inal and Kelleci (2012)	Turkey	1 hospital	IV (n = 123), E: 61, C: 62	6–12 (E:9.4 ± 2.1, C:9.3 ± 1.8)	Yes	No	RCT	Visual distraction (distraction cards)	Routine care	Pain: CAPS (0–5), FPS (0–10), anxiety	12
27	Jibb et al. (2018)	Canada	1 hospital	SCP needle insertion for chemo (EMLA 60 min) (n = 39), E: 18, C: 21	4–9 (E:6.3 ± 1.4, C:6.1 ± 1.5)	Yes	Yes	RCT	Web-based service cognitive-behavioral program (MEDiPORT humanoid robot)	Active distraction robot with dancing and singing	Pain: FPS, NRS (0–10), fear: CFS (0–10), distress: BAADS (1–5)	9
28	Kristja and Kristja (2011)	Canada	1 school	Immunization (n = 77), E: 38, C: 39	13–15 (14 ± 0.18)	Yes	No	RCT	Audial distraction (musical distraction: With headphone)	Standard care	Pain: VAS (0–10), anxiety, fear (0–10)	7
29	Kristja and Kristja (2011)	Canada	1 school	Immunization (n = 80), E: 41, C: 39	13–15 (14 ± 0.18)	Yes	No	RCT	Audial distraction (musical distraction: without headphone)	Standard care	Pain: VAS (0–10), anxiety, fear (0–10)	7
30	Nilsson et al. (2015)	Sweden	3 school	HPV vaccination (n = 37), E: 37, C: 37	11–12	Yes	Yes	Crossover RCT	Visual distraction (relaxation and guided imagery)	Standard care	Pain: CAS (0–10), FAS (0.04–0.97), salivary cortisol	10
31	Oliveira et al. (2016)	Brazil	1 hospital	IV (n = 40), E: 22, C: 18	6–12 (E:8.3 ± 2.1, C:8.7 ± 1.8)	Yes	No	Crossover RCT	Audiovisual distraction (Disney movie)	Routine care	Pain: VAS (0–10), FPS (0–10), PCS-C (13 item, 1–5), CSS (35 item, 0–4)	8
32	Hartling et al. (2013)	Canada	1 hospital	IV (n = 42), E: 21, C: 21	3–11	Yes	Yes	RCT	Audial distraction (musical distraction: with headphone)	Standard care	Pain: FPS-R (0–10), OSBD-R, anxiety	10
33	Redfern et al. (2018)	USA	1 hospital	Vaccination (n = 50), E: 25, C: 25	3–18 (E:10.7 ± 4.7, C:10.5 ± 4.7)	Yes	No	RCT	Visual distraction (distraction cards)	No intervention	Pain: WB-FACES, parent satisfaction (0–10)	12
34	Risaw et al. (2017)	India	1 hospital	Phlebotomy (n = 210), E: 105, C: 105	4–6 (4.8 ± 0.8)	Yes	No	RCT	Visual distraction (distraction cards)	Standard care	Pain: FLACC (0–10)	10
35	Bergomi et al. (2018)	Italy	1 hospital	IV (n = 75), E: 36, C: 39	5–12 (E:8.3 ± 2.2, C:9.4 ± 2.3)	Yes	No	RCT	Touch distraction (BUZZY device)	Standard care	Pain: WB-FPS, CEMS, parent anxiety: NRS (0–10)	7
36	Bergomi et al. (2018)	Italy	1 hospital	IV (n = 76), E: 37, C: 39	5–12 (E:9.4 ± 2.0, C:9.4 ± 2.3)	Yes	No	RCT	Visual distraction (cartoon)	Standard care	Pain: WB-FPS, CEMS, parent anxiety: NRS (0–10)	7
37	Bergomi et al. (2018)	Italy	1 hospital	IV (n = 77), E: 38, C: 39	5–12 (E:8.6 ± 2.1, C:9.4 ± 2.3)	Yes	No	RCT	Touch distraction (BUZZY device + cartoon)	Standard care	Pain: WB-FPS, CEMS, parent anxiety: NRS (0–10)	7
38	Meiri et al. (2016)	Israel	1 hospital	IV (n = 66), E: 33, C: 33	2–10 (E:5.4 ± 2.6, C:5.5 ± 2.6)	Yes	Yes	RCT	Medical clown (medical clown)	Standard care	Pain: VAS (0–10), anxiety (0–10), crying time	7
39	Meiri et al. (2016)	Israel	1 hospital	IV (n = 67), E: 34, C: 33	2–10 (E:5.0 ± 2.4, C:5.5 ± 2.6)	Yes	Yes	RCT	Local anesthesia (EMLA cream, 50min)	Standard care	Pain: VAS (0–10), anxiety (0–10), crying time	7
40	Karakaya and Duygu G€ozen (2016)	Turkey	1 hospital	IV (n = 144), E: 72, C: 72	7–12	Yes	No	RCT	Visual distraction (kaleidoscope)	No intervention	Pain: FPS (0–10), pulse oximeter, thermometer	7
41	Cerne et al. (2014)	Italy	1hospital	Immunization (n = 35), E: 18, C: 17	6	Yes	No	RCT	Audiovisual distraction (cartoon movie)	Standard care	Pain: WB-FACES Distress: OSBD-A	8
42	Vagnoli et al. (2015)	Italy	1hospital	Phlebotomy (n = 50), E: 25, C: 25	4–11 (E:7.1 ± 1.8, C:7.38 ± 2.5)	Yes	No	RCT	Visual distraction (dog presence)	Standard care	Pain: WB-FACES Distress: OSBD-A	10
43	Singh (2012)	India	1hospital	Immunization (n = 60), E: 30, C: 30	1.5–2	No	No	Quasi	Touch distraction (toy)	Standard care	Pain: FLACC (0–10)	5
44	Singh (2012)	India	1hospital	Immunization (n = 60), E: 30, C: 30	1.5–2	No	No	Quasi	Audial distraction (musical distraction)	Standard care	Pain: FLACC (0–10)	5
45	Minute et al. (2012)	Italy	1hospital	Immunization (n = 97), E: 47, C: 50	4–10 (median: 7)	Yes	No	RCT	Visual distraction (Wii videogame+ EMLA cream)	EMLA cream	Pain: FLACC (0–10), FPS-R	9

Notes. E: experimental group, C: control group, IV: intravenous, SCP: subcutaneous port, EMLA: eutectic mixture of local anesthetics, HPV: Human papillomavirus, IRB: institutional review board, RCT: randomized controlled trial, Quasi: quasi-experimental design, PSP: playstation portable, Ditto-D: ditto distraction, Ditto-PP: ditto procedural preparation, Ditto-C: ditto combined procedural preparation and distraction, RA: research assistant, WB-FACES: Wong Baker faces pain rating scale., CFS: children’s fear scale, NRS: numerical rating scale, VAS: visual analogue scale, FPS-R: face pain scale—revised, OSBD-R: observational scale of behavioral distress—revised, FLACC: face, legs, activity, crying and consolability behavioral scale, PRCD: parent report of child distress, MBPS: modified behavior pain scale, MAISD: measure of adult and infant soothing and distress, CAPS: children’s anxiety and pain scales, FPS: faces pain scale, BAADS: behavioral approach-avoidance scale, CAS: colored analogue scale, PCS-C: pain catastrophizing scale for children, CSS: child stress scale, WB-FPS: Wong-Baker faces pain rating scale, CEMS: children’s emotional manifestation scale, OSBD-A: amended observation scale of behavioral distress.

**Table 2:** The subgroup analysis of the effect of distract intervention on needle-related pain of the child by the study characteristics (N = 41).

Characteristics	Subgroup	K	N	Overall ES	95% CI		Z (*p*)	I^2^ (%)
					Lower Limit	Upper Limit		
Age (year)	<10	15	1419	0.27	−0.18	0.73	1.17 (0.242)	95.4
	≥10	26	1761	−1.07	−1.60	−0.53	−3.92 (<0.001)	85.3
Center	One	38	2671	−0.51	−0.85	−0.17	−2.96 (0.003)	92.7
	Multi-center	3	509	−0.43	−0.96	0.09	−1.61 (0.108)	0.0
IRB	No	4	233	1.97	−4.31	8.25	0.61 (0.539)	98.7
	Yes	37	2947	−0.64	−0.89	−0.39	−5.04 (<0.001)	84.9
Fund	Yes	15	1340	−0.83	−1.20	−0.46	−4.41 (<0.001)	90.5
	No	26	1840	−0.18	−0.84	0.47	−0.55 (0.581)	93.0
Study design	RCT	37	2932	−0.61	−0.92	−0.30	−3.85 (<0.001)	91.3
	Quasi-E	4	248	0.94	−3.60	5.48	0.40 (0.685)	96.9
Type of intervention	Audial	7	394	−0.38	−2.10	1.33	−0.44 (0.661)	94.9
	Visual	17	1790	−0.76	−1.15	−0.37	−3.86 (<0.001)	89.6
	Clown	2	119	−3.64	−4.76	−2.52	−6.36 (<0.001)	91.9
	Cognitive	2	107	−0.47	−1.64	0.70	−0.79 (0.428)	0.0
	Multi methods	12	703	0.31	−0.35	0.97	0.92 (0.360)	93.4
	Local anesthesia	1	67	0.67				
Quality assessment score	<10	24	2077	−0.35	−1.16	0.46	−0.84 (0.400)	93.6
	≥10	17	1103	−0.65	−0.98	−0.32	−3.84 (<0.001)	89.3

Notes. K: Number of studies, N: Number of participants, ES: Effect size, CI: Confidence interval, IRB: Institutional Review Board, RCT: Randomized controlled trial, Quasi-E: Quasi-experimental design.

**Table 3:** The effect of distract intervention on distress of the Child.

Variables	K	N	Overall ES	95% CI		Z (*p*)	I^2^ (%)
				Lower Limit	Upper Limit		
Observer pain (parent)	23	1891	−0.40	−1.26	0.45	−0.93 (0.355)	94.1
Observer pain (nurse)	28	2173	1.43	−1.96	4.82	0.83 (0.408)	99.7
Child-reported anxiety	3	207	−0.33	−1.27	0.62	−0.68 (0.496)	0.0
Observer anxiety (parent)	15	1163	−0.68	−1.13	−0.23	−2.96 (0.003)	85.9
Observer anxiety (nurse)	14	1330	−1.03	−1.67	−0.40	−3.20 (0.001)	94.0
Cortisol	5	340	−1.11	−2.09	−0.12	−2.20 (0.274)	66.8

## Supplementary Materials

The following are available online at https://www.mdpi.com/article/10.3390/ijerph18179159/s1, Table S1. Search Strategy for Databases.
