# Supplementary material for: Effect of Distraction Intervention for Needle-Related Pain and Distress in Children: A Systematic Review and Meta-Analysis
Source: Int J Environ Res Public Health. 2021 Aug 31;18(17):9159. doi: 10.3390/ijerph18179159 (PMC8430753; doi:10.3390/ijerph18179159)
Supplement: Supplementary file 1 [file ijerph-18-09159-s001.zip › ijerph-1349789-supplementary.pdf]

Supplementary Table S1. Search Strategy for Databases.

Database	Search strategies	Results
PubMed	Search: **((CHILD) AND (DISTRACTION)) AND (PAIN)** Filters: **English, Korean, Child: birth-18 years, from 2011/1/1 - 2019/7/31** Sort by: **Most Recent**<br>(("child"[MeSH Terms] OR "child"[All Fields] OR "children"[All Fields] OR "child s"[All Fields] OR "children s"[All Fields] OR "childrens"[All Fields] OR "childs"[All Fields]) AND ("distract"[All Fields] OR "distractability"[All Fields] OR "distractable"[All Fields] OR "distracted"[All Fields] OR "distracter"[All Fields] OR "distracters"[All Fields] OR "distractibility"[All Fields] OR "distractible"[All Fields] OR "distracting"[All Fields] OR "distraction"[All Fields] OR "distractional"[All Fields] OR "distractions"[All Fields] OR "distractive"[All Fields] OR "distracts"[All Fields]) AND ("pain"[MeSH Terms] OR "pain"[All Fields])) AND ((2011/1/1:2019/7/31[pdat]) AND (english[Filter] OR korean[Filter]) AND (allchild[Filter]))<br>**Translations**<br>**CHILD:** "child"[MeSH Terms] OR "child"[All Fields] OR "children"[All Fields] OR "child's"[All Fields] OR "children's"[All Fields] OR "childrens"[All Fields] OR "childs"[All Fields]<br>**DISTRACTION:** "distract"[All Fields] OR "distractability"[All Fields] OR "distractable"[All Fields] OR "distracted"[All Fields] OR "distracter"[All Fields] OR "distracter's"[All Fields] OR "distracters"[All Fields] OR "distractibility"[All Fields] OR "distractible"[All Fields] OR "distracting"[All Fields] OR "distraction"[All Fields] OR "distractional"[All Fields] OR "distractions"[All Fields] OR "distractive"[All Fields] OR "distracts"[All Fields]<br>**PAIN:** "pain"[MeSH Terms] OR "pain"[All Fields]	371
	Search: **((child or children) AND (distraction intervention)) AND (needle-related pain or venipuncture pain or immunization pain)** Filters: **from 2011/1/1 - 2019/7/31** Sort by: **Most Recent**	85
Embase	Search: ('**child**'/exp OR '**child**') AND ('**distraction**'/exp OR '**distraction**') AND ('**pain**'/exp OR '**pain**') AND ([controlled clinical trial]/lim OR [randomized controlled trial]/lim) AND [2011-2019]/py	106
	Search: ('children'/exp OR 'children') AND 'distraction intervention' AND 'needle-related pain' OR 'venipuncture pain' OR 'immunization pain' AND (2011:py OR 2012:py OR 2013:py OR 2014:py OR 2015:py OR 2016:py OR 2017:py OR 2018:py OR 2019:py) AND 'article'/it AND ([adolescent]/lim OR [child]/lim OR [infant]/lim OR [preschool]/lim OR [school]/lim) AND ('controlled clinical trial'/de OR 'human'/de OR 'randomized controlled trial'/de)	27
CINAHAL	Search: 'CHILD' AND 'DISTRACTION' AND 'PAIN' Filters: Full Text; Peer-Reviewed; 20110101-20190731; English	88
	Search: 'children' AND 'distraction intervention' AND ('needle-related pain' OR 'venipuncture pain' OR 'immunization pain') Filters: Full Text; Peer-Reviewed; 20110101-20190731; English	9
